# Retention of metals in periprosthetic tissues of patients with metal-on-metal total hip arthroplasty is reflected in the synovial fluid to blood cobalt transfer ratio in the presence of a pseudotumour

**DOI:** 10.1186/s12891-020-03636-0

**Published:** 2020-09-12

**Authors:** Tomi Nousiainen, Sanna Palosaari, Sirpa Peräniemi, Arja Tervahauta, Jaakko Niinimäki, Juhana Leppilahti, Petri Lehenkari

**Affiliations:** 1 Medical Faculty, Cancer and Translational Medicine Research Unit, University of Oulu and Medical Research Center, Oulu University Hospital, P.O. Box 5000, 90014 Oulu, Finland; 2 Division of Orthopaedic Surgery, Oulu University Hospital, Oulu, Finland; 3 University of Eastern Finland, School of Pharmacy, P.O. Box 1627, 70210 Kuopio, Finland; 4 Department of Environmental and Biological Sciences, University of Eastern Finland, P.O. Box 1627, 70210 Kuopio, Finland; 5 Division of Radiology, Oulu University Hospital, Oulu, Finland

**Keywords:** Metal-on-metal hip, Pseudotumour, Adverse reaction, Metals, Revision surgery

## Abstract

**Background:**

Modern metal-on-metal (MOM) arthroplasties were performed for over a decade before alarming reports of adverse metal reactions dramatically reduced their use. Failures are seen more often with high-wearing implants, but also well-positioned components with more favourable wear patterns can cause problems. There are no specific clinical indicators that could help us to predict the prognosis of these implants. For this reason, we still need more information on the effect of underlying factors that contribute to this process.

**Methods:**

In this prospective cohort study, we investigated how cup orientation and type of pseudotumour determined by the Hart classification effect the distribution of metals in blood, synovial fluid and tissues surrounding the metal-on-metal hip prosthesis in revision surgery patients. One thousand two hundred twenty-nine metal-on-metal hip patients were screened and of those, 60 patients that had a revision surgery due to adverse metal reaction were included. Whole blood, synovial fluid and synovial/pseudotumour tissue samples were analysed for metal ion concentrations (Co, Cr, Mo and Ti).

**Results:**

The lowest metal concentrations were found when both cup anteversion and inclination were optimal, and the highest when both were suboptimal. Suboptimal anteversion alone raised Cr-ion concentrations more than suboptimal inclination. The concentrations of metals in blood, synovial fluid or synovial soft tissue were the same in patients with and without a pseudotumour, but the relative transfer percentage of cobalt from synovial fluid to blood was higher in patients with a pseudotumour.

**Conclusions:**

The implant orientation alone does not explain the metal concentrations found in tissues or distribution of metals between different tissues. The accumulation of metals in periprosthetic soft tissues increase the total metal load, and in the presence of a pseudotumour this is reflected in the transfer ratio of Co from synovial fluid to the blood. The total metal load of the pseudotumour tissue should be defined in future studies to determine if this will provide new insights for clinical practice.

## Background

During recent years there has been extensive research on metal-on-metal (MOM) hip prostheses because of the increasing awareness of adverse reactions. The clinical aspects of MOM implants have been studied widely and the follow-up protocols and clinical practices have evolved through years of research and clinical experience. There are studies which explain these reactions by local toxic and hypersensitivity reactions to wear particles [1–6]. Implant malpositioning has been suggested as the cause for the rise in blood metal ion levels [7] and implant properties as the reason for wear [8, 9]. Adverse reactions have also been detected when the implant is in an optimal position [10]. The properties and size of wear particles from the articular surface or taper junctions have been found to differ on a nanometer scale, and it is suggested [11] that particle properties could be more important than total particle burden in the occurrence and severity of adverse tissue reactions. Still, there is concern about possible systemic toxicity caused by these wear particles that are disseminated into the periprosthetic tissues and also to remote locations in the body [12].

The common denominator for these studies is that adverse reactions are found more often than anticipated, and clinical manifestations vary [13–15]. The awareness of implant reactions might partially explain the rise, but there is substantial evidence [16–20] that this is a real clinical phenomenon, although some of these reactions can be asymptomatic. It is unsettling that there are no specific clinical indicators that could predict the prognosis of these prostheses. It is also unknown why so many patients with abnormal metal reactions remain clinically painless until devastating soft tissue lesions progress and cause limping and disability [21].

The terminology of these adverse reactions varies (ALVAL, aseptic lymphocyte-dominated vasculitis-associated lesion; ARMD, adverse reaction to metal debris; ALTR, adverse local tissue reaction) but the term pseudotumour is universally used. Different follow-up protocols are advised, but individual parameters like blood ions, plain radiographs or clinical symptoms in isolation can be misleading and should therefore be used in combination [22]. It has been shown that the best accuracy is achieved by measuring blood ions and MARS MRI (metal artifact reduction sequence magnetic resonance imaging) regularly [23]. Even though implantation of MOM prostheses has almost ceased, we must bear in mind the economical aspect of follow-ups, considering the immense number of patients with these implants globally. For this reason, we need more information on the mechanical, physiological and cellular events behind the adverse effects.

The aim of this study is to describe the effect of acetabular component angles and pseudotumour formation on the blood metal ion levels, one of the clinical indicators of implant wear.

## Methods

### Patients

**Fig. 1:**
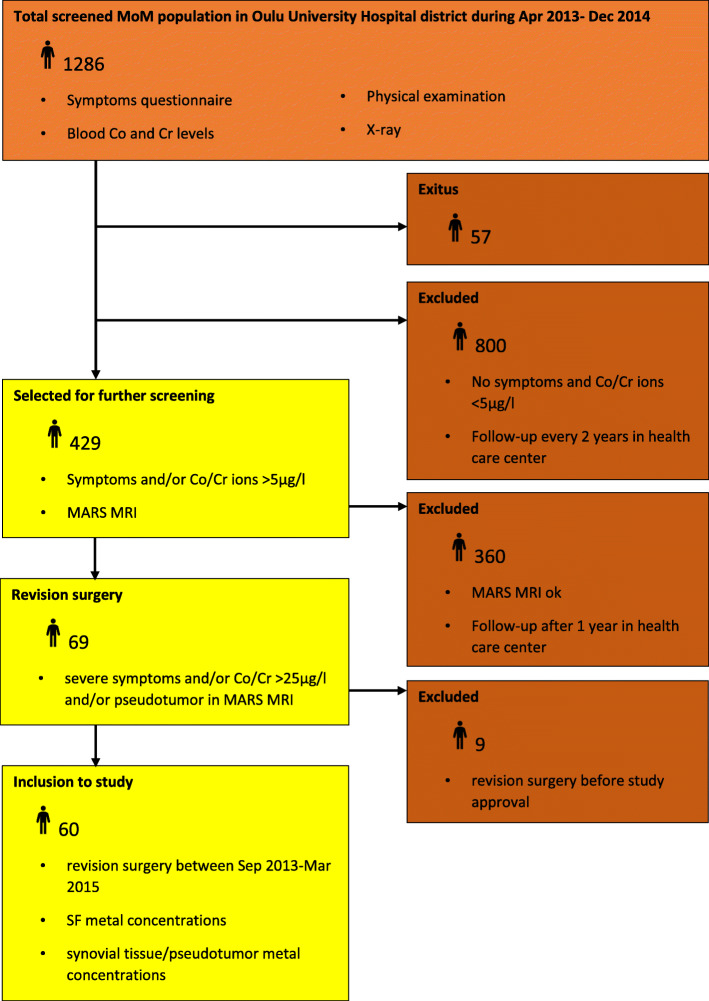
Inclusion criteria

This prospective cohort study was approved by the Ethics Committee of Oulu University Hospital, Oulu, Finland, and complies with the Declaration of Helsinki. All patients gave their written informed consent. A flow chart of inclusion criteria of this study is shown in Fig. 1 and the detailed screening protocol is presented in Supplement S1.

Between April 2013 and December 2014, a total of 1286 patients with MOM hip arthroplasty were screened in Oulu University hospital. Of those, 429 patients were symptomatic or had ion levels more than 0.005 ppm (5 μg/l). These were further examined by MARS MRI. A total of 69 hips were revised between September 2013 and March 2015, due to the results of MARS MRI, patient symptoms and blood ion levels. The study began after the first nine revisions, and a total of 60 patients were included to the study.

Sixty primary total hip arthroplasties were performed between March 1997 and May 2011 (Table 1.). The indication for total hip arthroplasty was end-stage osteoarthritis (OA) in 41 (68.3%) cases, rheumatoid arthritis in 9 (15%) cases, trauma in 6 (10%) cases and secondary OA in 4 (6.7%) cases. The mean age of patients at the time of primary hip implantation was 55 years (SD 10.4) and at the time of MOM revision 63 years (SD 9.8). Several different primary implants (Table 2) were used, but the most common was the Bimetric/M2A38 combination. The mean in-situ time of MOM implants was 86 months (SD 24.1).

**Table 1:** Demographic data of included patients

Variables	Value
Gender	
Female	35 (58%)
Male	25 (42%)
Side	
Right hip	41 (68%)
Left hip	19 (32%)
Age at the time of revision, years	
Mean	63 (SD 9.8)
Range	Min 30 – max 80
Implant in-situ time, months	
Mean	86 (SD 24.1)
Range	Min 29 – max 132
BMI, kg/m^2^	
Mean	28.9 (SD 4.9)
Range	Min 17.5 – max 38.6
Weight, kg	
Mean	80.9 (SD 16.4)
Range	Min 46 – max 130
Cup anteversion, degrees	
Mean	21.6 (SD 10.9)
Range	Min −7 – max 46
Cup inclination, degrees	
Mean	49.9 (SD 9.5)
Range	Min 26 – max 70
WB Co, μg/l	
Mean	29.6 (SD 37.7)
Range	Min 0.8 – max 193.2
WB Cr, μg/l	
Mean	13.4 (SD 16.3)
Range	Min 0.5 – max 95.9
Oxford Hip Score, points	
Mean	35.8 (SD 13.4)
Range	Min 12 – max 48

**Table 2:** Different implant types

Implant type	Number of implants	Head size (mm)
Bimetric/M2A38 (Biomet)	38	38
Bimetric M2A Magnum/Recap (Biomet)	11	Median 46 (40–54)
CDH/M2A38 (Biomet)	1	38
Integral/M2A38 (Biomet)	1	38
Profemur T/Conserve (Wright Medical)	1	42
ML Taper/Durom (Zimmer)	1	44
Corail/Pinnacle (DePuy)	1	36
Anthology/R3 (Smith&Nephew)	1	40
Synergy/BHR Modularis (Smith&Nephew)	1	54
Taperloc M2A Magnum/Recap (Biomet)	1	N/A
Recap resurfacing (Biomet)	2	42 and 48
BHR resurfacing (Smith&Nephew)	1	46

MARS MRI was performed on 57 (95%) patients. There were three patients for whom MRI was not performed, one patient had a cardiac pacemaker, one patient had a dorsal root nerve stimulator and one patient had severe symptoms requiring revision. Clinical findings during revision were abnormal in all those three cases. MRI findings were evaluated using the classification described by Hart [10, 24]. Abnormal MRI findings were Hart 1 in 8 (14%) cases, Hart 2A in 14 (24.6%) cases, Hart 2B in 12 (21.1%) cases and Hart 3 in 9 (15.8%) cases. MRI findings were normal on 14 (24.6%) cases. Patients who had more than one tumour (n 20) were classified by the more severe grade.

Version and inclination of acetabular components were measured from standard pelvic x-ray pictures which were obtained from our digital Picture Archiving and Communication System (PACS). One of the authors (TN) made all the measurements. Inclination was measured from standard anteroposterior pelvic X-rays using a digital angle meter and reference lines were drawn from the lowest point of the pelvic rami and edges of the acetabular component. This is found to be a reliable and accurate method [25]. Version was measured from lateral cross-Table X-rays with the same method and using reference lines from the edges of the acetabular component and a line perpendicular to the horizontal plane. This method is described by Woo and Morrey [26].

### Revision surgery

All revisions were carried out at Oulu University Hospital. Posterior approach was used in all revisions, and when removal of a well-fixed femoral component was needed, an extended proximal femoral osteotomy was used as described by Younger [27]. An average of three culture samples were taken (range 1–6) from the hip joint to rule out infection. In 57 cases all cultures were clean and the remaining three patients had not had previous infection. In two cases we found one positive enrichment culture after 10 days, one patient with *Propionibacterium acnes* in 1/3 cultures and another patient with *Staphylococcus warneri* in 1/4 cultures. Neither of these patients had infection before, during or after the MOM revision. These cultures were deemed to have been contaminations. One patient without previous signs of infection gave two positive enrichment cultures (10 days) out of five with *Staphylococcus capitis.* He developed a clinical infection after a few weeks latent time. The infection was treated with revision surgery and antibiotics.

During revisions a joint fluid aspirate was taken (synovial fluid, SF, n 53) before opening the deep fascia. There were 7 cases with a dry joint cavity. Biopsies from the pseudotumour tissue, or from neo-synovium if a pseudotumour was not present (soft tissue, ST, n 60), were taken after opening the joint. Two soft tissue samples were not available for metal analyses. Whole blood (WB, n 60) samples were also collected and analysed.

The clinical findings during revision varied greatly, ranging from a tight fibrous capsule around the hip joint to a bulky, sack-like pseudotumour around the hip. The appearance of joint fluid ranged from greyish to black metallic to milky white. In two cases femoral stems were loose. Acetabular components were loose in five cases, all of which were M2A38. In four patients, joint fluid contained rice-like debris. Taper of the femoral component was mentioned to be good in 18 (31,6%) cases, discolored, dark or tarnished in 29 (50,9%) cases and worn in 4 (7%) cases. Total hip revision was done in nine (15%) cases including all HRAs (hip resurfacing arthroplasties) and the remaining 51 (85%) cases were cup revisions.

### Metal analysis

For whole blood Co and Cr analyses, samples were collected from the patient’s antecubital vein in heparin-containing trace element blood tubes. To avoid metal contamination from the needle, the standard protocol for blood sample collection for these analyses included rinsing of the collection needle by collecting one spare tube prior to actual sample collection. Quantification of blood Co and Cr concentrations were conducted by Vita Laboratoriot using the clinically accredited inductively coupled plasma mass spectrometry (ICP-MS) method.

A soft tissue sample adjacent to the synovial cavity, or if present from the synovial pseudotumour, was collected into a dry container and frozen at − 80 °C until analysed. For metal analysis, 0.1 g samples were weighed with a Mettler Toledo MX5 (readability 0.1 mg), placed in a teflon digestion vessel and 8.0 ml HNO_3_ (TraceMetal Grade, Fisher Chemical, A509-P1) was added. The samples were microwave digested using a MARS 6 iWave instrument and an Animal Tissue method. After the digestion, the samples were diluted to 20 ml with de-ionized water (USF Elga Maxima). The concentrations of the analytic elements were measured with an ICP-MS (Perkin Elmer NexION 350D) using the kinetic energy discrimination (KED) mode. The instrument was calibrated with multielement standards (TraCERT Periodic table mix 1 for ICP, Sigma-Aldrich, 92,091 and TraCERT Periodic table mix 2 for ICP, Fluka, 41,135) and a single element standard for Hg (CertiPUR, Merck, 170,226). Synovial soft tissue samples from three patients undergoing primary hip replacement surgery were used as controls reflecting metal concentrations of synovial soft tissue that has not been exposed to a metal-implant.

For metal analyses of synovial fluid (100 μl), 2.0 ml HNO_3_ (TraceMetal Grade, Fisher Chemical, A509-P1) was added and the samples were digested using ultrasound assisted acid digestion. After 15 min the digestion sample was diluted to 5 ml with de-ionized water (USF Elga Maxima). The concentrations of the analytic elements were measured with ICP-MS (Perkin Elmer NexION 350D) using the KED mode. The instrument was calibrated with multielement standards (TraCERT Periodic table mix 1 for ICP, Sigma-Aldrich, 92,091 and TraCERT Periodic table mix 2 for ICP, Fluka, 41,135) and a single element standard for Hg (CertiPUR, Merck, 170,226). Synovial fluid samples from three patients undergoing primary hip replacement surgery were used as controls reflecting metal concentrations of synovial soft tissue that has not been exposed to a metal-implant.

The distribution of metals was analysed in relation to the presence or absence of pseudotumours. The SF and ST Cr and Co concentrations were proportioned to blood Cr and Co concentrations by dividing the WB concentration by the SF or ST concentration and converting these to percentages.

### Statistical analyses

SPSS for Windows (IBM Corp. Released 2018. IBM SPSS Statistics for Windows, Version 25.0. Armonk, NY: IBM Corp.) was used for data analysis. For total metal concentrations the mean values (with standard deviation) are reported. A non-parametric Kruskal-Wallis test for independent samples was used for the evaluation of differences in metal concentrations between groups defined by implant cup angles (anteversion optimal/inclination optimal, AO/IO; anteversion optimal/inclination suboptimal, AO/IS; anteversion suboptimal/inclination optimal, AS/IO; anteversion suboptimal/inclination suboptimal, AS/IS) and pseudotumour type (no tumour, Hart 1, Hart 2A, Hart 2B, Hart 3). The Mann-Whitney test was used for evaluation of differences between metal concentrations (WB, SF and ST Co and Cr) and between percentual transfer ratios from SF or ST to blood in patients with or without a pseudotumour. The results are given as median with 25th–75th percentiles. *P*-values were adjusted by the Bonferroni correction for multiple tests and the values < 0.05 were considered statistically significant.

## Results

### Quantification of selected metal ions

Synovial fluid and synovial soft tissue samples were measured for Co, Cr, Mo and Ti. Only Co, Cr and Mo were found consistently in samples and thus were further analysed statistically. Titanium concentrations exceeding the mean + 2SD of controls were found in 18 synovial fluid and 13 soft tissue samples (Supplement S2).

**Fig. 2:**
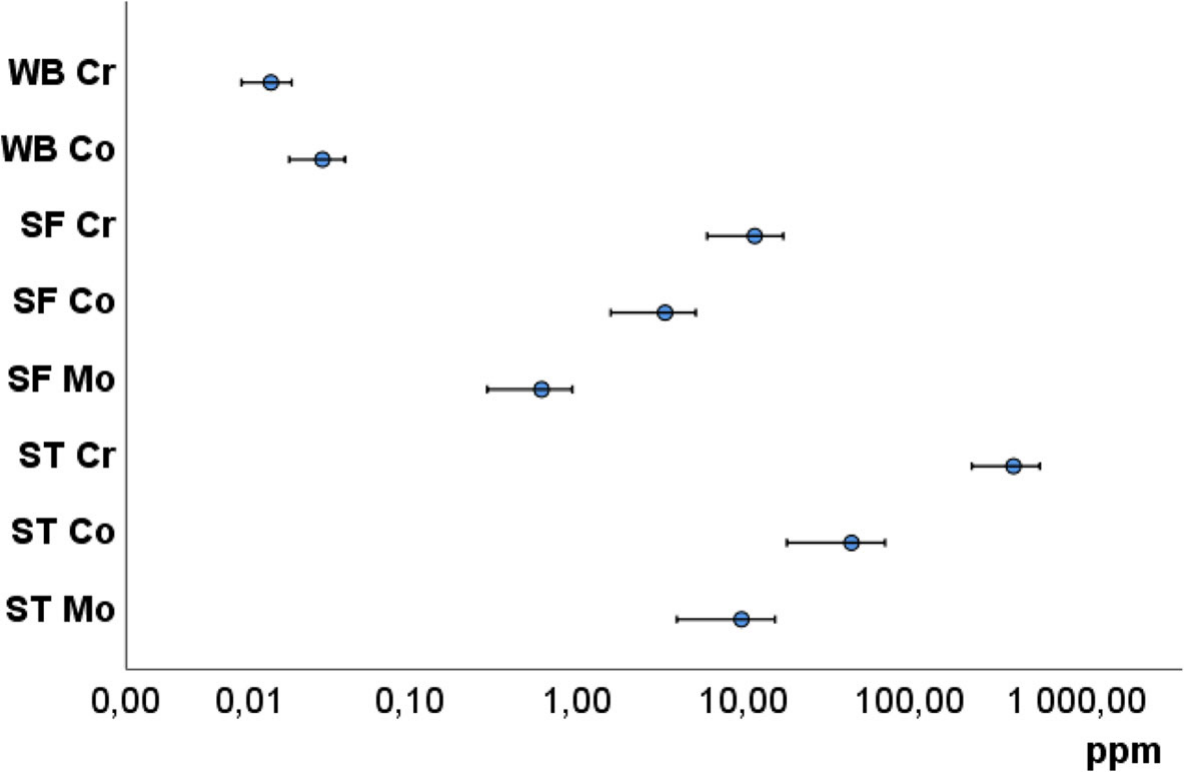
Metal concentrations in whole blood (WB), synovial fluid (SF) and synovial soft tissue (ST). The highest local metal concentrations were found in the synovial soft tissue followed by synovial fluid and blood. The distribution of chromium was different between tissues showing local accumulation of chromium in the synovial soft tissue

The metal concentrations in the studied tissues are presented in Fig. 2. In blood, the Cr concentration median was 0.0053 (25–75 percentile range 0.022) ppm and Co median 0.0079 (25–75 percentile range 0.046) ppm. No other metals were measured from these samples. In synovial fluid of MOM patients, Cr concentrations were 312-fold higher and Co concentrations 115-fold higher than in their blood. In controls, metal concentrations were negligible. Cr concentrations of MOM patients’ soft tissues were 10,476-fold and Co concentrations 1325-fold higher than in their blood.

We tested but did not find any statistically significant differences in metal ion levels (whole blood, synovial fluid, synovial tissue/pseudotumour) in relation to BMI, weight, height, implant type, implant head size or the implant in situ time.

### Effect of implant angles on metal concentrations

**Fig. 3:**
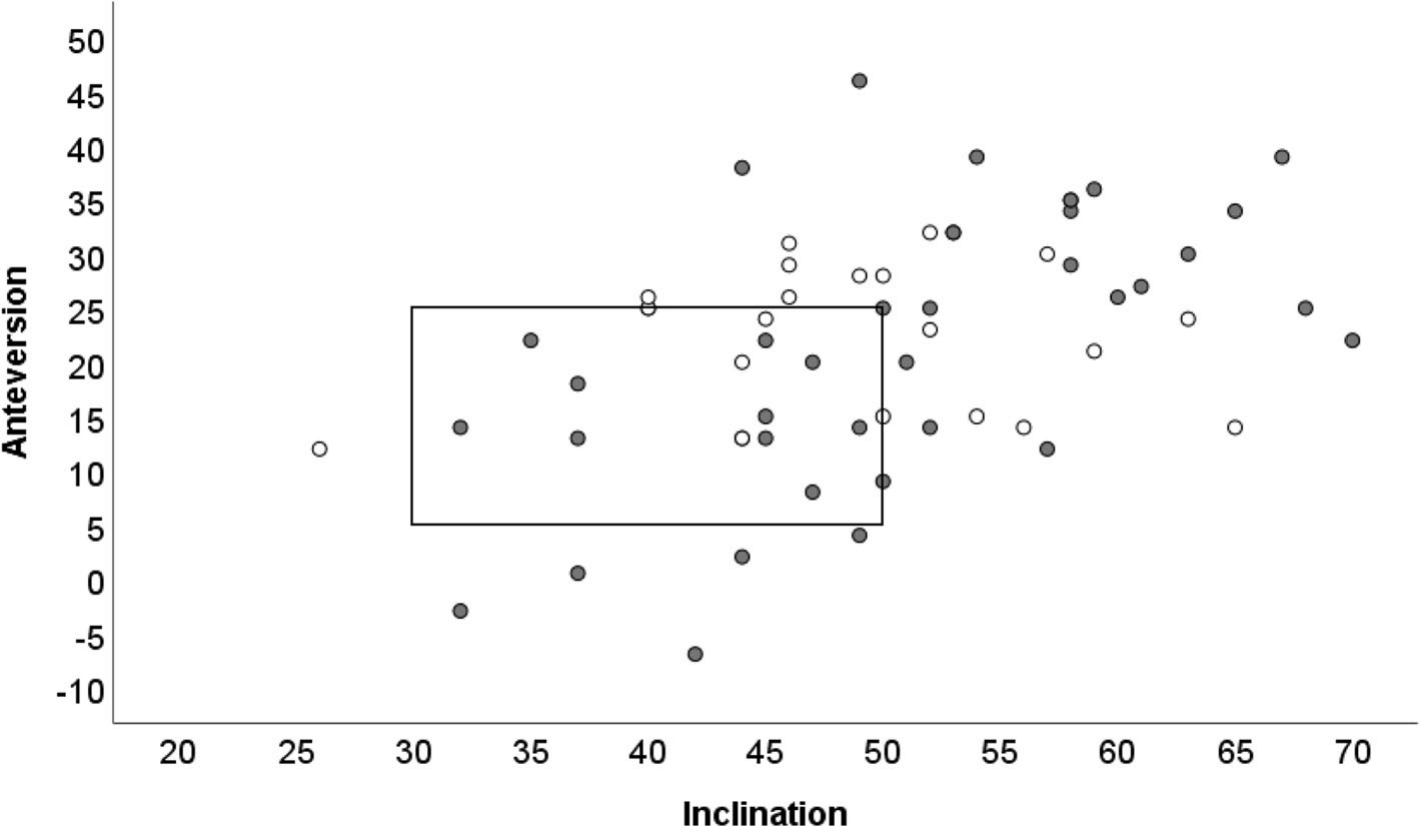
The distribution of anteversion and inclination angles in the MOM revision patients included in this study. Lewinnek safe zone [28] is indicated by a square and the patients with WB Cr and Co concentrations ≤0.005 ppm (5 μg/l) are marked with white dots and patients with Cr and/or Co concentrations ≥0.005 ppm (5 μg/l) are marked with gray dots

We found a great diversity regarding the orientation of acetabular components. Only one third of cups were implanted correctly in the safe zone as defined by Lewinnek, anteversion 15+/− 10° and inclination 40+/− 10° [28] (Fig. 3). Fifty-five percent (n 33) of implants had correct anteversion and 55% (n 33) had correct inclination. Cup orientation was correct in both planes in 33.3% (n 20). 20% (n 12) of cups were out of the safe zone in both planes. Most (n 16/21) of those patients who had low ≤0.005 ppm (≤ 5 μg/l) WB Co and WB Cr concentrations had poor cup orientation either in both planes or only poor anteversion or inclination. Inside the safe zone 15 of 20 patients had elevated ≥0.005 ppm (≥ 5 μg/l) WB Co and/or Cr ions.

**Fig. 4:**
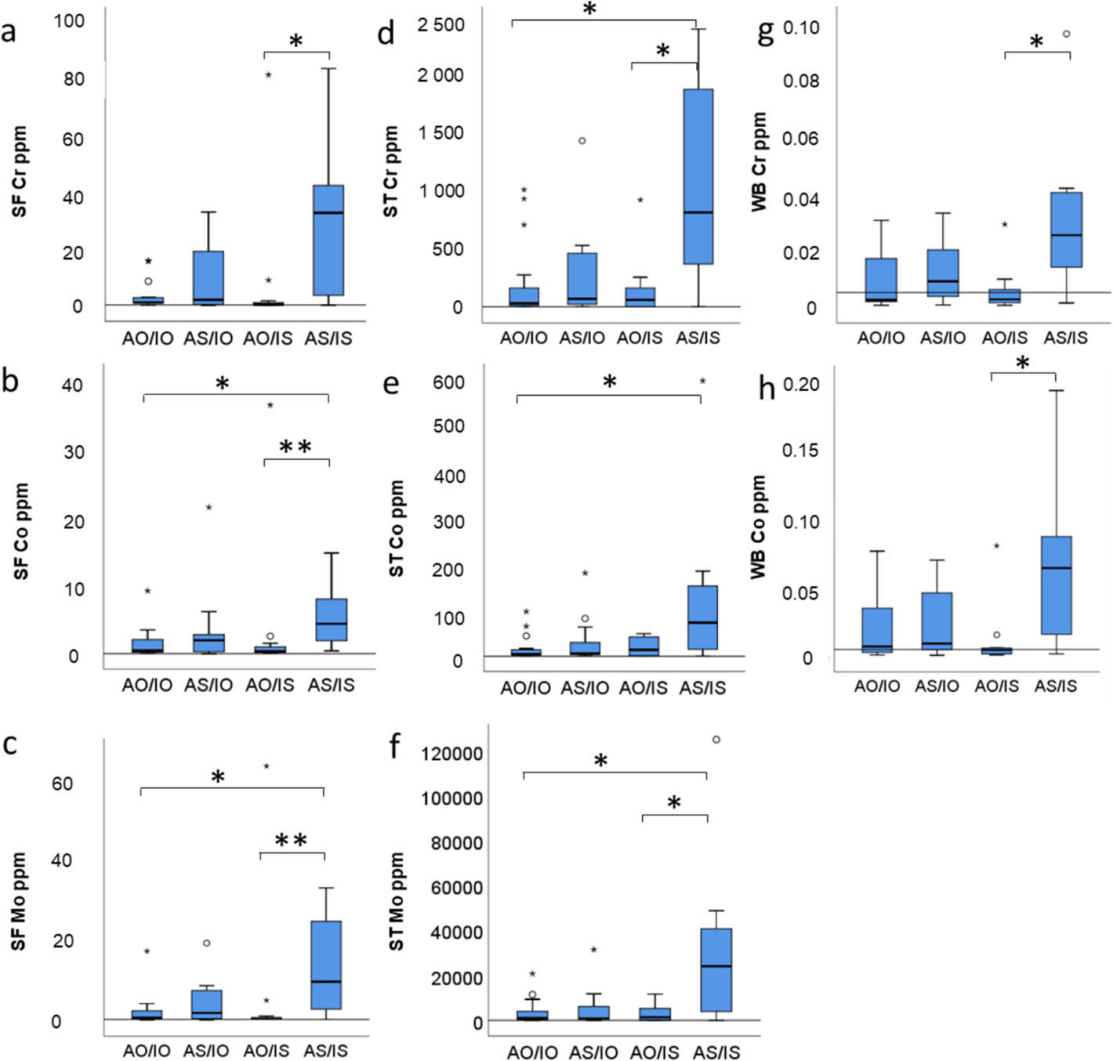
Effect of anteversion and inclination angles on metal concentrations. Mean + 2SD of controls is indicated by a horizontal line for synovial fluid (SF) and soft tissue (ST) samples. AO, anteversion optimal; AS, anteversion suboptimal; IO, inclination optimal; IS, inclination suboptimal. For SF and ST: AO/IO n 15, AS/IO n 13, AO/IS n 11, AS/IS n 11; For WB: AO/IO n 19, AS/IO n 15, AO/IS n 13, AS/IS n 13; * *p* < 0.05, ** *p* < 0.01

**Fig. 5:**
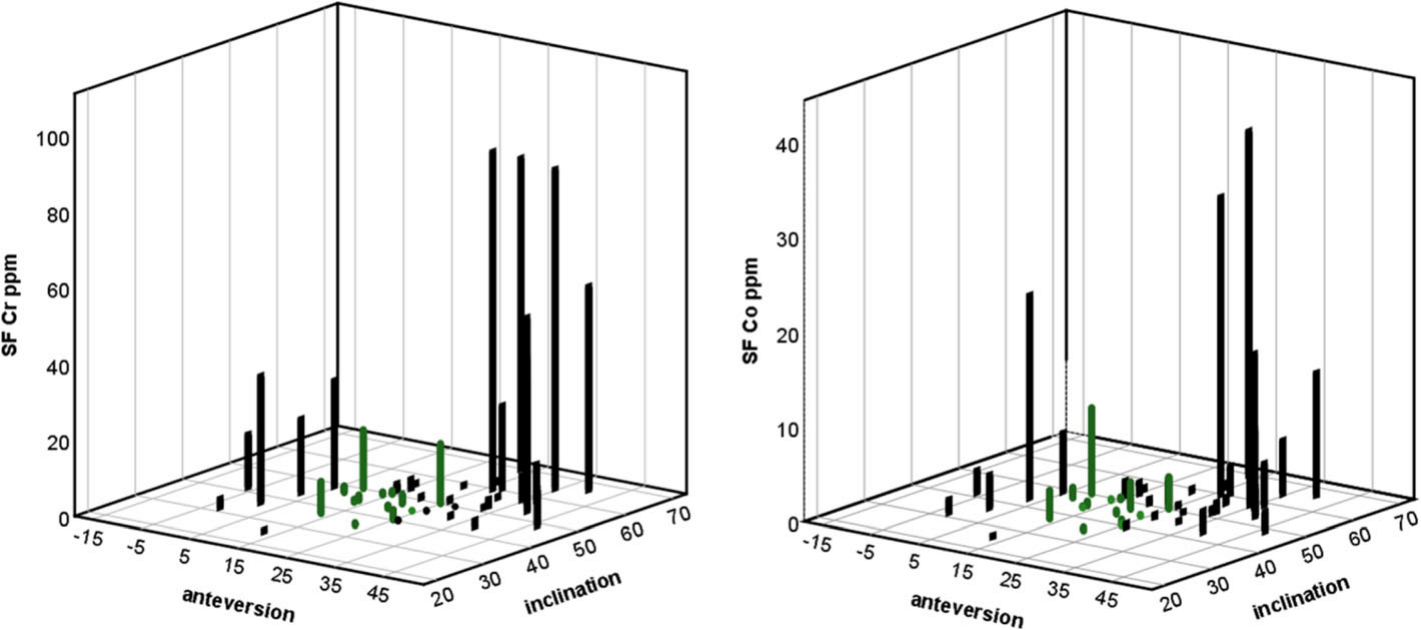
3D graph showing concentration of synovial fluid Cr and Co in relation to anteversion and inclination angles. Cases with optimal implant angles are presented with green bars

Metal concentrations in all tissues were lowest when both anteversion and inclination angle were in the optimal range and highest when both were suboptimal (Fig. 4 and Table 3) Suboptimal anteversion angle alone raised Cr concentrations in all tissues more than suboptimal inclination alone (Fig. 4a, d, g; Fig. 5; Supplements S3 and S4). Statistically significant differences between groups were found between suboptimal inclination (AO/IS) and malorientation in both planes (AS/IS) (Table 3). When the cup was malorientated in both planes, the Cr concentration increased approximately 99-fold in SF (*p* = 0.015), 14-fold in ST (*p* = 0.016) and 8-fold in WB (*p* = 0.018); Co increased 13-fold in SF (*p* = 0.006) (Fig. 4b) and in WB (*p* = 0.032) (Fig. 4h) and Mo increased 32-fold in SF (*p* = 0.008) (Fig. 4c) and 16-fold in ST (*p* = 0.027) (Fig. 4f). The metal concentrations were also significantly increased when suboptimal orientation in both planes (AS/IS) were compared to optimal orientation in both planes (AO/IO). Here the increase of Co concentration was 9-fold in SF (*p* = 0.030), 17-fold in ST (*p* = 0.011) (Fig. 4b, e, f). The increase of Mo concentration was 16-fold in SF (*p* = 0.037) and 22-fold in ST (*p* = 0.017) (Fig. 4c, f) and Cr concentration increased 28-fold in ST (*p* = 0.017) (Fig. 4d).

**Table 3:** Distribution of metals (median with 25th–75th percentiles) in tissues by anteversion and inclination angles

	AO/IO (n 20)	AS/IO (n 13)	AO/IS (n 13)	AS/IS (n 14)
**Synovial fluid**	**(ppm)**	**(ppm)**	**(ppm)**	**(ppm)**
**Cr**	**1.10**	**2.01**	**0.33**	**32.78**
(*p* = 0.020)*	(0.77–2.85)	(0.35–20.35)	(0.18–1.67)	(4.91–49.24)
**Co**	**0.46**	**1.95**	**0.33**	**4.36**
(*p* = 0.006)**	(0.23–2.95)	(0.25–3.08)	(0.16–1.54)	(2.40–9.24)
**Mo**	**0.06**	**0.18**	**0.03**	**0.95**
(*p* = 0.008)**	(0.03–0.32)	(0.03–0.73)	(0.02–0.10)	(0.34–2.78)
**Soft tissue**				
**Cr**	**30.00**	**70.00**	**60.00**	**830.00**
(*p* = 0.013)*	(10.00–280.00)	(15.00–505.00)	(0.00–180.00)	(552.50–2115.00)
**Co**	**4.30**	**5.50**	**13.60**	**72.70**
(*p* = 0.012)*	(2.40–16.70)	(2.25–46.30)	(0.600–43.20)	(13.52–179.95)
**Mo**	**1.10**	**1.00**	**1.50**	**24.20**
(*p* = 0.012)*	(0.40–5.20)	(0.45–7.95)	(0.10–5.70)	(4.82–45.42)
**Whole blood**				
**Cr**	**0.002**	**0.009**	**0.003**	**0.025**
(*p* = 0.024)*	(0.001–0.023)	(0.003–0.021)	(0.001–0.007)	(0.019–0.042)
**Co**	**0.007**	**0.009**	**0.005**	**0.064**
(*p* = 0.029)*	(0.002–0.035)	(0.004–0.047)	(0.002–0.006)	(0.022–0.097)

*p*-values from Kruskal-Wallis test, * *p* < 0.05 and ** *p* < 0.01

### Effect of pseudotumour on distribution of metals

The concentration of metals (Cr and Co) in different tissues (WB, SF, ST) did not differ statistically significantly between patients with or without a pseudotumour (Supplementary file S5). The percentage of Co transferred from SF to WB was 1.7-fold higher in patients with than without a pseudotumour (*p* = 0.039) (Table 4). No differences in the percentage of Co transferred from ST to WB or Cr transferred from SF or ST to WB were found (Table 4).

**Table 4:** The relative percentage (median with 25th–75th percentiles) of Cr and Co transferred from SF or ST to WB

	No tumour	Tumour
**WB/SF**	**%**	**%**
**Cr**	**0.207**	**0.265**
(*p* = 0.304)	(0.073–0.598)	(0.160–0.592)
**Co**	**0.667**	**1.154**
(*p* = 0.039)	(0.358–1.322)	(0.683–2.003)
**WB/ST**		
**Cr**	**0.003**	**0.005**
(*p* = 0.741)	(0.002–0.032)	(0.002–0.008)
**Co**	**0.066**	**0.071**
(*p* = 0.892)	(0.021–0.427)	(0.039–0.239)

**Fig. 6:**
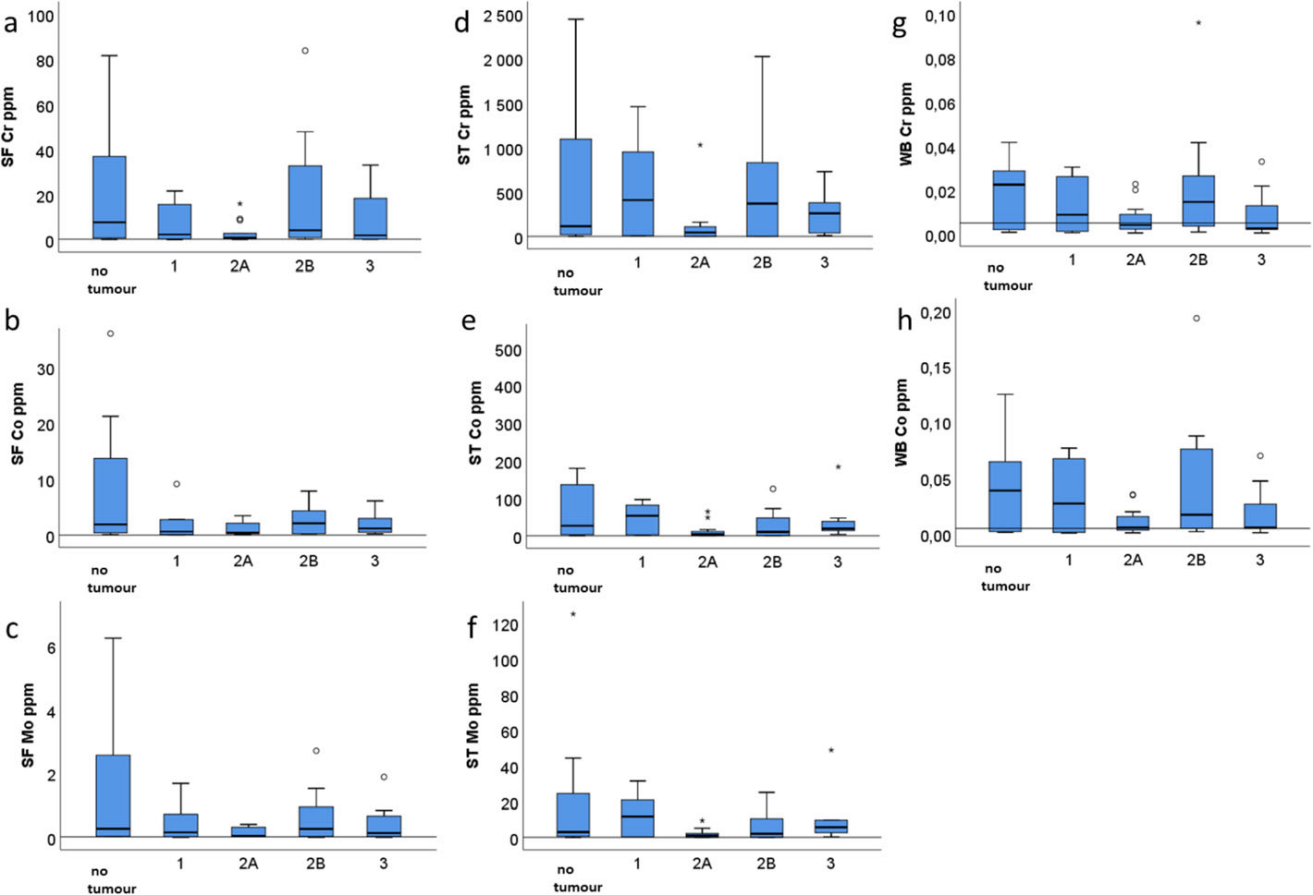
Distribution of metals by Hart classification [24] of pseudotumours. The highest synovial fluid (SF) and whole blood (WB) metal concentrations were found in patients without a pseudotumour, and lowest in patients with a fluid-filled type 2A pseudotumour. The lowest whole blood metal concentrations were found in patients with type 3 pseudotumours. The lowest soft tissue (ST) metal concentrations were in patients with type 2A tumour and highest in 1, 2B, and 3 tumours, suggesting retention of metals in the pseudotumour tissue. SF: no tumour n 13, 1 n 6, 2A n 14, 2B n 10, 3 n 7; ST: no tumour n 12, 1 n 8, 2A n 14, 2B n 12, 3 n 9; WB: no tumour n 14, 1 n 8, 2A n 14, 2B n 12, 3 n 9. Reference line is set to mean + 2SD of controls for SF and ST and to 0.005 ppm (5 μg/l) for WB

Patients with type 2A pseudotumours had lower overall metal concentrations in SF, ST and blood when compared to other groups. The levels of Cr, Co and Mo in synovial fluid (Table 4. and Fig. 6a-c) were higher in patients without pseudotumours than in patients with pseudotumours. The metal concentrations in blood (Fig. 6g, h) followed the trend of the metal concentrations in the synovial fluid as Cr and Co concentrations were highest in patients without pseudotumours and lowest in patients with type 2A and 3 pseudotumours. The distribution of metals in the soft tissue samples (Fig. 6d-f) differed from the synovial fluid and blood. The highest ST Cr concentrations were in patients with type 1 (4-fold higher compared to no tumour) and type 2B tumours (3-fold higher), followed by patients with type 3 tumours (2-fold higher), suggesting retention of Cr in the pseudotumour tissue (Fig. 6d). The distribution of Co and Mo in soft tissue (Fig. 6e, f) was different from Cr, as the highest Co concentrations were found in patients with type 1 tumours (2-fold higher compared to no tumour) and up to 7-fold lower in patients with other types of tumours. Mo concentration was highest in patients with type 1 tumours (4-fold higher compared to no tumour) and showed great variation between groups. Despite the observed differences in the metal concentrations between groups, the differences did not reach statistical significance (Table 5).

**Table 5:** Distribution of metals (median with 25th–75th percentiles) in tissues by Hart classification [24] of pseudotumours

	No tumour (n 14)	Hart 1 (n 8)	Hart 2A (n 14)	Hart 2B (n 12)	Hart 3 (n 9)
**Synovial fluid**	**(ppm)**	**(ppm)**	**(ppm)**	**(ppm)**	**(ppm)**
**Cr**	**7.69**	**2.24**	**0.85**	**4.12**	**1.88**
(*p* = 0.308)	(0.51–37.02)	(0.23–17.11)	(0.29–4.22)	(0.78–36.56)	(0.22–27.62)
**Co**	**1.91**	**0.62**	**0.38**	**2.11**	**1.18**
(*p* = 0.202)	(0.35–14.23)	(0.07–4.38)	(0.21–2.33)	(0.24–4.66)	(0.40–3.39)
**Mo**	**0.28**	**0.16**	**0.06**	**0.27**	**0.14**
(*p* = 0.327)	(0.03–2.79)	(0.02–0.97)	(0.02–0.33)	(0.03–1.10)	(0.02–0.85)
**Soft tissue**					
**Cr**	**115.00**	**410.00**	**45.00**	**370.00**	**260.00**
(*p* = 0.463)	(15.00–1172.50)	(10.00–1077.50)	(0.00–120.00)	(0.00–1072.50)	(20.00–470.00)
**Co**	**26.85**	**53.50**	**3.80**	**10.40**	**19.40**
(*p* = 0.197)	(2.55–157.40)	(2.10–85.48)	(0.35–12.80)	(0.50–53.88)	(11.40–47.50)
**Mo**	**3.05**	**11.75**	**1.10**	**2.10**	**5.70**
(*p* = 0.328)	(0.50–30.85)	(0.38–23.55)	(0.10–2.33)	(0.10–13.93)	(0.90–9.80)
**Whole blood**					
**Cr**	**0.022**	**0.008**	**0.004**	**0.015**	**0.003**
(*p* = 0.142)	(0.002–0.029)	(0.001–0.027)	(0.002–0.009)	(0.003–0.030)	(0.002–0.022)
**Co**	**0.039**	**0.027**	**0.006**	**0.017**	**0.006**
(*p* = 0.085)	(0.002–0.073)	(0.001–0.070)	(0.003–0.017)	(0.005–0.078)	(0.005–0.047)

*p*-values from Kruskal-Wallis test

## Discussion

In this study, we focused on the distribution of metals in periprosthetic tissues, synovial fluid and systemic circulation in relation to implant orientation and the presence or absence of a pseudotumour.

We first determined the overall metal load in the tissues. The most abundant metals found in the tissues of MOM patients were Cr, Co and Mo, as expected and reported in the literature. The processing of Cr and Co seems to differ in the body [29, 30]; Co is removed from tissues and Cr shows retention. This is shown by the higher relative concentration of Co compared to Cr in blood, but in other tissues the relationship is the reverse. Some samples (synovial tissue n 13 and synovial fluid n 18) contained also elevated levels of titanium, which plausibly originates from the femoral taper. This hypothesis is supported by the fact that 18/33 cases of our study population with tarnished, discolored or worn femoral taper showed also elevated titanium levels in ST and/or SF.

Next, we looked at the effect of the implant orientation on the release of metal debris. We found great diversity regarding the orientation of acetabular components in the enrolled patients. Only 1/3 of cups were implanted correctly in the safe zone as defined by Lewinnek [28]. This finding was in line with our previous work [31] where we found that cup orientation was good in 41.5% of the total MOM population and only in 30.8% of patients with clinical indication for revision. Many studies [7, 8] have shown that poor component orientation increases the risk for an adverse reaction after hip arthroplasty. It has been suggested that inclination angle affects the blood metal concentration more than the anteversion angle [7]. Our data is in agreement with the hypothesis that the inclination angle shows a more linear relationship to blood metal concentrations compared to anteversion angle (Supplements S3 and S4), but mostly because the malposition in inclination does not settle on both sides of the Lewinnek’s safe zone, as it does with anteversion. This means that there is a tendency for inclination that is too steep, but we seldom see cups that are too horizontal. Instead version is quite often either slightly retroverted or too steeply anteverted [7, 8, 31]. When both orientations were taken into account, we found that poor anteversion alone led to higher ion levels when compared to poor inclination alone (Figs. 4 and 5). The highest metal ion levels were found when the cup orientation was poor in both planes. Adverse reactions have been reported also in well-functioning and well-positioned hips [10, 24]. This can be partially due to the fact that not only the wear particles from the articulating surface, but also modular junctions between the components either on the femoral or acetabular side can contribute to particle load in soft tissues [32]. In the current study we did not measure the orientation of the femoral components, which affect the total component orientation and implant wear. This study shows that even with optimal component orientation, the whole blood Co and/or Cr ion levels may be elevated. Furthermore, whole blood Co and Cr ion levels can be low even though the implant orientation is poor. These observations highlight the inaccuracy of individual clinical parameters in predicting the implant survival.

Finally we focused on local factors that could affect the distribution of metals. In the presence of a pseudotumour the percentage of cobalt transferred from synovial fluid to whole blood was higher than in patients without a tumour. Since there were no differences between the groups in the SF or WB Co concentrations, there must be another source for the observed higher Co transfer. The pseudotumour retains some of the metals and adds to their transition to the circulation. It is known that cobalt is more readily transported to the blood and excreted in the urine, but chromium is accumulated in tissues [29, 33]. This is shown in the percentual transfer ratio of Co and explains why Co concentrations were higher than Cr in blood and the reverse was true for synovial fluid and soft tissue. This finding is important in the clinical perspective, since blood sampling is the standard method for ion level monitoring. In liquid samples (WB, SF) the measured concentration accurately reflects the absolute amount of metal in the tissue. In soft tissue biopsies, however, the sample may reflect only focal changes. We found interesting observational differences between the subgroups of pseudotumours. Soft tissue metal concentrations were higher in patients with type 1, type 2B or type 3 tumours and blood concentrations were highest in patients without a tumour (Fig. 6). Although there were differences in metal concentrations between the Hart classes, the differences were not statistically significant. Therefore, these pseudotumour subtype analyses should be interpreted as experimental and hypothesis generating. There are previous studies examining the correlation between whole blood, synovial fluid or synovial tissue metal ion levels and histological findings or cellular parameters. Reito et al. [34] studied the possible diagnostic utility of joint fluid metal ion measurement for histological findings, but neither cobalt nor chromium were found to have good predictive value. Lehtovirta et al. [35] found that whole blood, joint fluid and synovial tissue metal concentrations correlated poorly with histological findings. Yet, to our knowledge there are no previous studies examining the role of pseudotumours in the distribution of metals. In our opinion, this observation deserves to be investigated also in a larger cohort of patients. Previous studies, including our current study, examined only focal biopsies of the synovial/pseudotumour tissue, which may not reflect the total synovial metal load. The analysis of the whole pseudotumour, showing the total metal load, could give more accurate information and possibly show a stronger correlation with the blood concentrations.

There were also 14 patients without a radiologically detectable pseudotumour in this study. Clinical findings of the hip joint were abnormal in all these patients during the revision. Osteolysis was found in eight (57.1%) patients and component loosening in three (21.4%) patients. Most of the patients (71.4%) had elevated blood metal ion levels and the vast majority (78.6%) of cups were implanted poorly either in regard to anteversion or inclination or in both planes. Also, the metal concentrations in synovial fluid were highest in patients without a pseudotumour. It can be argued that these implants had increased wear rates because of poor implantation. Such elevated metal burden locally has cytotoxic effects which can cause tissue destruction. This can be evaluated with MARS MRI but diagnostics should not be limited only to ruling out pseudotumours.

There are some shortcomings in this study. First, the sample size (n 60) is relatively small and the patients with the most severe symptoms (n 9) were not included since they were operated before the study approval. Second, the selection of patients for this study was based solely on the clinical indications for revision. This led to a situation where the samples were from patients with different types of prostheses that can have different patterns of wear, which can increase variation in the results. However, we did not find statistically significant differences in metal ion levels and the implant head size or implant type. Furthermore, the heterogeneity of the samples reflects the actual situation that is faced in orthopaedic practice. Third, we acknowledge the inaccuracy of plain radiographs compared to cross-sectional imaging when evaluating cup position. Yet, pelvic x-rays are readily available and commonly used by orthopaedic surgeons. Also, there are several different classifications of pseudotumours and interobserver reliability of their identification varies. In a recent study [36], the Hart classification that we used was found to have better interobserver reliability than the classifications by Anderson [37] or Hauptfleisch [38].

These observations show the importance of systematic screening of MOM patients, including appropriate imaging and subsequent surgical intervention. Also, if revision surgery is performed the surrounding periprosthetic tissues should be thoroughly analysed histologically and biologically to provide more detailed information of this pathology.

## Conclusions

This study, in agreement with earlier findings, shows the heterogeneous results of individual screening parameters, which should never be evaluated in isolation. High tissue metal load can be seen in patients with poorly implanted components. However, adverse tissue reactions are found even in patients with optimal implant orientation. While interpreting the results of our study, it should be noted that the study population was selected based on the need for revision surgery. Periprosthetic soft tissue shows retention of metals, increasing the patients’ total metal load. This is reflected at least in the WB/SF Co percentual transfer ratio. Due to our small sample size it is hard to formulate any solid and generalized recommendations, but multidisciplinary screening of these patients is mandatory. Future studies with thorough analysis of periprosthetic soft tissues may be able to reveal new markers to better understand these destructive processes.

## Supplementary information

**Additional file 1.** Detailed screening protocol.

**Additional file 2.** Ti concentrations exceeding mean + 2SD of controls were found in 18 MOM synovial fluid samples and in 13 soft tissue samples.

**Additional file 3.** Metal concentrations in synovial fluid (SF), soft tissue (ST) and whole blood (WB) in relation to anteversion angle.

**Additional file 4.** Metal concentrations in synovial fluid (SF), soft tissue (ST) and whole blood (WB) in relation to inclination angle.

**Additional file 5.** Metal concentrations in synovial fluid (SF), soft tissue (ST) and whole blood (WB) in relation to presence or absence of a pseudotumour.

## Data Availability

All data generated or analysed during this study are included in this published article and its supplementary information files.

## Abbreviations

ALTR: Adverse local tissue reaction
ALVAL: Aseptic lymphocyte-dominated vasculitis-associated lesion
AO: Anteversion optimal
ARMD: Adverse reaction to metal debris
AS: Anteversion suboptimal
BMI: Body mass index
HRA: Hip resurfacing arthroplasty
IO: Inclination optimal
IS: Inclination suboptimal
MARS MRI: Metal artifact reduction sequence magnetic resonance imaging
MOM: Metal-on-metal
PACS: Picture archiving and communication system
SF: Synovial fluid
ST: Synovial tissue
WB: Whole blood
