# Supplementary material for: Retention of metals in periprosthetic tissues of patients with metal-on-metal total hip arthroplasty is reflected in the synovial fluid to blood cobalt transfer ratio in the presence of a pseudotumour
Source: BMC Musculoskelet Disord. 2020 Sep 12;21:610. doi: 10.1186/s12891-020-03636-0 (PMC7488743; doi:10.1186/s12891-020-03636-0)
Supplement: Supplementary file 1 — Additional file 1. Detailed screening protocol. [file 12891_2020_3636_MOESM1_ESM.docx]

**Supplement S1.** Detailed screening protocol.

1.) All patients filled a questionnaire regarding possible symptoms. Whole blood cobalt (Co) and chromium (Cr) concentrations were analysed with ICP-MS (inductively coupled plasma mass spectrometry). Pelvic X-rays were taken and clinical examination was done.

If the patient was symptomless and the whole blood ion levels were below 5µg l^-1^, then the patient would have their next control after 2 years with the same examination.

2.) Those patients that were symptomless but whose ion levels (either Co or Cr) were above 5 µg l^-1^ were screened with MARS MRI. If the MRI was normal, then the next control would be after 1 year, when the clinical examination, x-rays and whole blood ion analysis would be repeated. MRI would be repeated if ion levels increased. On the other hand, if we found a pseudotumour in MRI, a revision arthroplasty was done.

3.) Those patients who had symptoms or there was an abnormal sound of the hip implant, were screened with MARS MRI. If the MRI was normal, the next control was carried out after 1 year as above. If a pseudotumour was found in MRI, a revision arthroplasty was done.

4.) Highly elevated (above 25 µg l^-1^) ions prompted MARS MRI to be done. With highly elevated ion levels the risk of adverse reaction to metal debris is high. For these patients we suggested revision even if the MRI was normal.

Our first line screening showed that two-thirds (n=800/1229) of patients were asymptomatic and Co and Cr ions were low (below 5 µg l^-1^). One-third (n=429/1229) of patients had symptoms and/or raised ion levels (above 5 µg l^-1^). Of those patients, the vast majority (n=360/429, 84%) had a normal MRI and follow-ups were to be continued. ARMD was found only in 16% (n=69/429) of patients with symptoms or elevated ion levels. Those patients were booked for revision. During first line screening, an adverse metal reaction was found in 5.6% of this MOM population. There were nine revisions done because of very severe symptoms before we got the approval from the Ethics Committee, and those patients were not included in this study.
