# Supplementary figures and images for: Retention of metals in periprosthetic tissues of patients with metal-on-metal total hip arthroplasty is reflected in the synovial fluid to blood cobalt transfer ratio in the presence of a pseudotumour
Source: BMC Musculoskelet Disord. 2020 Sep 12;21:610. doi: 10.1186/s12891-020-03636-0 (PMC7488743; doi:10.1186/s12891-020-03636-0)

## Slide 1
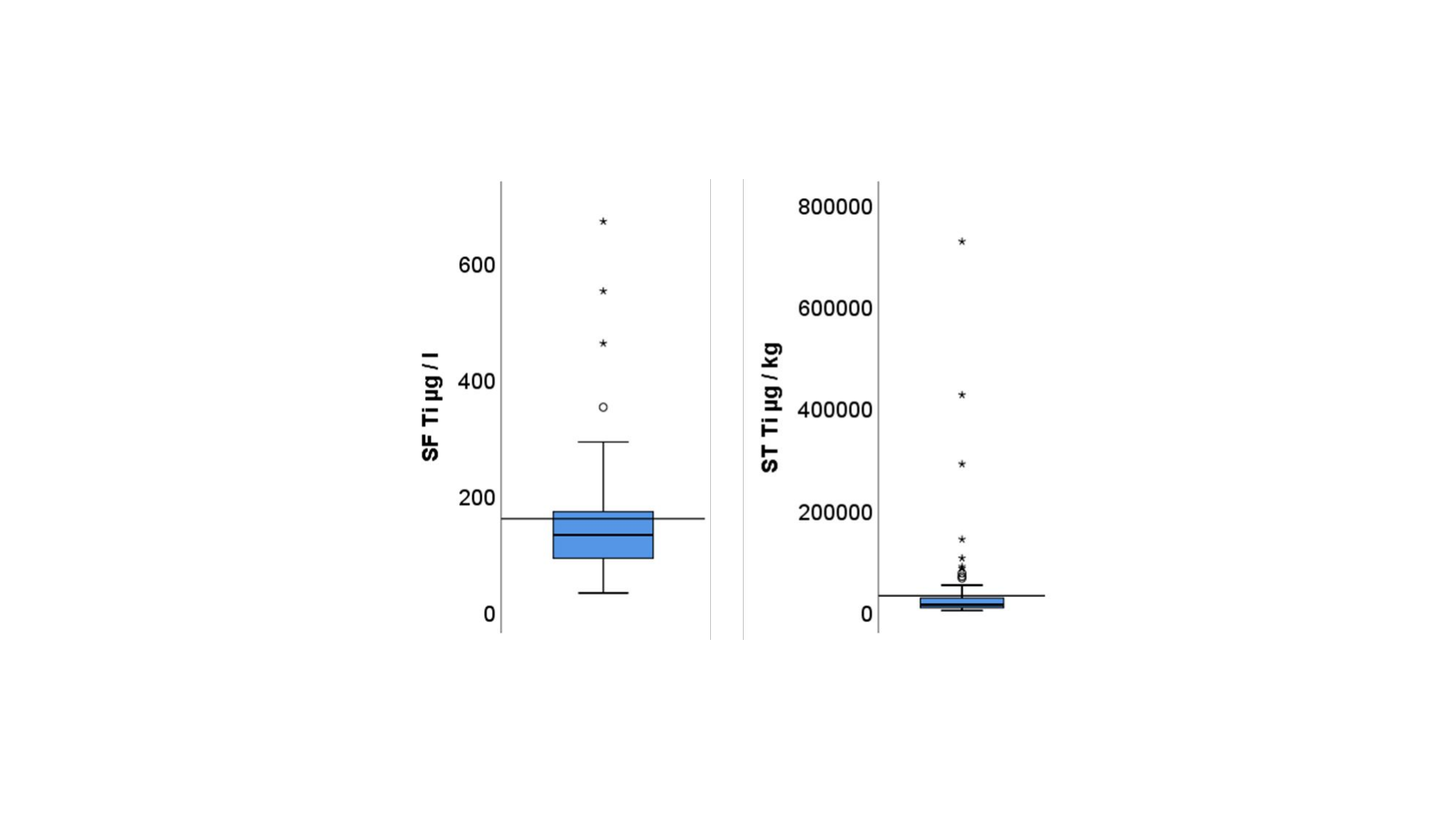

Supplement: Supplementary file 2 — Additional file 2. Ti concentrations exceeding mean + 2SD of controls were found in 18 MOM synovial fluid samples and in 13 soft tissue samples. [file 12891_2020_3636_MOESM2_ESM.pptx]

## Slide 1
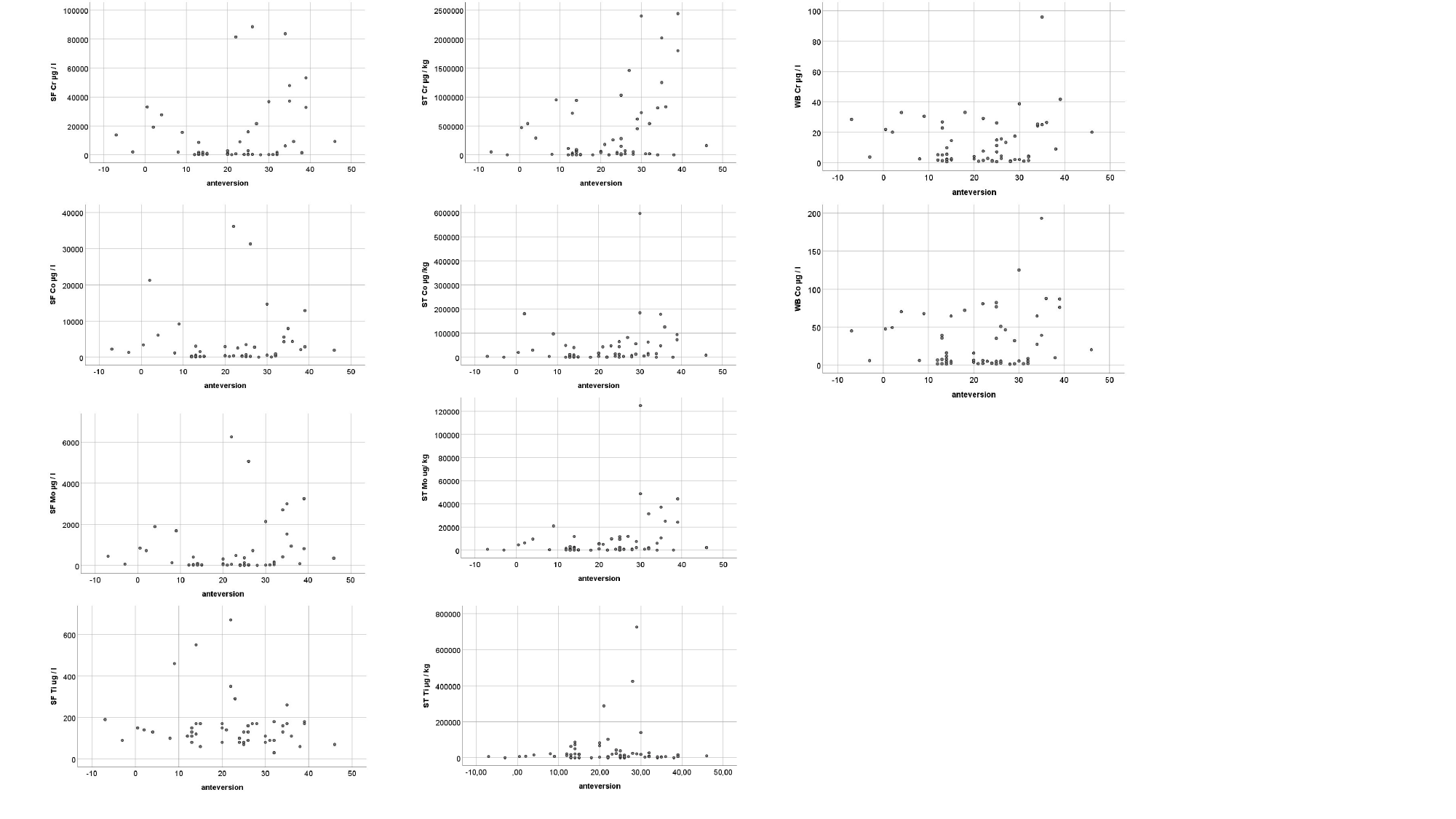

Supplement: Supplementary file 3 — Additional file 3. Metal concentrations in synovial fluid (SF), soft tissue (ST) and whole blood (WB) in relation to anteversion angle. [file 12891_2020_3636_MOESM3_ESM.pptx]

## Slide 1
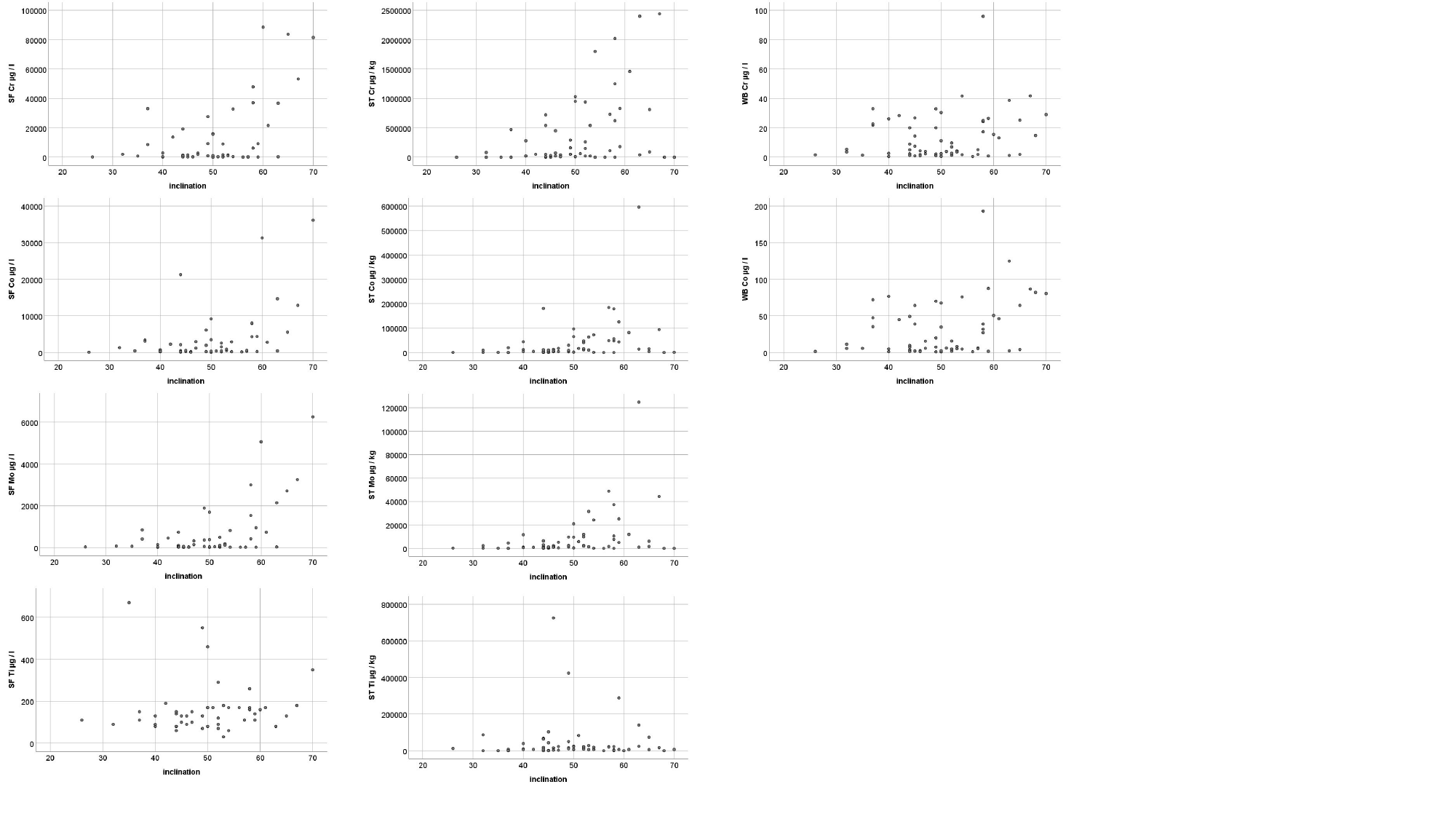

Supplement: Supplementary file 4 — Additional file 4. Metal concentrations in synovial fluid (SF), soft tissue (ST) and whole blood (WB) in relation to inclination angle. [file 12891_2020_3636_MOESM4_ESM.pptx]
