# Supplementary material for: Retention of metals in periprosthetic tissues of patients with metal-on-metal total hip arthroplasty is reflected in the synovial fluid to blood cobalt transfer ratio in the presence of a pseudotumour
Source: BMC Musculoskelet Disord. 2020 Sep 12;21:610. doi: 10.1186/s12891-020-03636-0 (PMC7488743; doi:10.1186/s12891-020-03636-0)
Supplement: Supplementary file 5 — Additional file 5. Metal concentrations in synovial fluid (SF), soft tissue (ST) and whole blood (WB) in relation to presence or absence of a pseudotumour. [file 12891_2020_3636_MOESM5_ESM.pdf]

	No tumour	Tumour
**WB**	**ppm**	**ppm**
**Cr**	**0.018**	**0.004**
(p= 0.119)	(0.002-0.029)	(0.002-0.022)
**Co**	**0.042**	**0.006**
(p= 0.151)	(0.002-0.068)	(0.003-0.046)
**SF**		
**Cr**	**13.71**	**1.43**
(p= 0.133)	(0.71-45.19)	(0.320-9.235)
**Co**	**2.27**	**0.69**
(p= 0.066)	(0.38-17.99)	(0.23-3.01)
**ST**		
**Cr**	**115.00**	**50.00**
(p= 0.346)	(15.00-1172.50)	(10.00-470.00)
**Co**	**26.85**	**8.40**
(p= 0.166)	(2.55-157.40)	(1.20-43.90)
